# Unveiling the significance of TREM1/2 in hemorrhagic stroke: structure, function, and therapeutic implications

**DOI:** 10.3389/fneur.2024.1334786

**Published:** 2024-02-07

**Authors:** Yancheng Kong, Di Wang, Xu Jin, Yi Liu, Hui Xu

**Affiliations:** ^1^Trauma Emergency Center, Changzhou Hospital of Traditional Chinese Medicine, Changzhou, China; ^2^Changzhou Hospital Affiliated to Nanjing University of Chinese Medicine, Changzhou, China

**Keywords:** TREM, intracerebral hemorrhage, subarachnoid hemorrhage, neuroinflammation, neuroprotection

## Abstract

Stroke has long been a major threat to human health worldwide. Hemorrhagic stroke, including intracerebral hemorrhage and subarachnoid hemorrhage, exhibits a high incidence rate and a high mortality and disability rate, imposing a substantial burden on both public health and the economy and society. In recent years, the triggering receptor expressed on myeloid cells (TREM) family has garnered extensive attention in various pathological conditions, including hemorrhagic stroke. This review comprehensively summarizes the structure and function of TREM1/2, as well as their roles and potential mechanisms in hemorrhagic stroke, with the aim of providing guidance for the development of targeted therapeutic strategies in the future.

## 1 Introduction

Stroke is the second leading global cause of death, the third major contributor to disability, and a primary cause of dementia (1). Its incidence is rising, regardless of whether one resides in a high-income or low-to middle-income country, particularly among the younger population (under 55 years). Projections suggest that the total cost of stroke will increase from $891 billion annually in 2017 to a staggering $23.1 trillion by 2050. Stroke can be categorized into ischemic stroke, which has a higher incidence rate, and hemorrhagic stroke, characterized by a significantly higher mortality and disability rate, making it more threatening (2, 3). Current treatment strategies for stroke are far from satisfactory. Recently, the Triggering Receptor Expressed on Myeloid cells (TREM)-1/2 has been discovered to play a significant role in both physiological and pathological processes (4), and its involvement in stroke has attracted widespread attention, as evidenced by a series of preclinical studies (5–8). The role of TREMs in ischemic stroke has been well-summarized and, therefore, is beyond the scope of this review (9–11). In this review, we have systematically outlined the structure and function of TREM1/2, as well as their roles and potential mechanisms in hemorrhagic stroke, with the aim of providing new insights into the development of potential therapeutic options.

## 2 Overview of hemorrhagic stroke

Hemorrhagic stroke comprises intracerebral hemorrhage (ICH) and subarachnoid hemorrhage (SAH). ICH refers to the rupture of blood vessels within the brain parenchyma, leading to the leakage of blood into adjacent brain tissue. ICH is typically caused by the spontaneous rupture of intracranial blood vessels, called spontaneous ICH (12). Additionally, factors such as trauma, tumors, vascular malformations, and the use of anticoagulants/fibrinolytic drugs can also lead to ICH, which is beyond the scope of this article. Spontaneous ICH is the most common form of hemorrhagic stroke, typically caused by hypertension and cerebral amyloid angiopathy. It carries a high mortality and disability rate, affecting approximately 2 million people globally each year (13). Gaining a deeper understanding of the pathological mechanisms of ICH is of paramount significance, as this will aid in developing targeted treatment approaches.

The injury mechanisms of ICH encompass both primary and subsequent secondary damage (14). Primary damage occurs within the initial hours after the onset of bleeding, primarily driven by the mass effect of the hematoma (15). Secondary damage refers to a cascade of reactions resulting from primary injury and the release of blood clot components, including excitotoxicity (16, 17), oxidative stress (18, 19), neuroinflammation (16, 18, 20, 21), disruption of the blood–brain barrier (BBB) (21, 22), ultimately leading to neuronal cell death (18, 23). Of these, neuroinflammation mainly involves early activation of resident microglia, release of pro-inflammatory mediators and influx of peripheral immune cells (24, 25). Stimulation of ICH acts on different microglia receptors including Toll-like receptors (TLRs) and the receptor of advanced glycosylation endproducts (26). TLRs, including TLR4, are involved in the neuroinflammatory process after ICH (27). TLR4 is predominantly expressed in CD11b + microglia and is upregulated early after ICH, followed by upregulation of pro-inflammatory genes through the nuclear factor-κB (NF-κB) signaling pathway (28). In addition to the above, blood components such as thrombin, fibrin and hemoglobin can trigger the inflammatory process via the TLR/NF-κB pathway (29, 30). Expression of adhesion molecules on leukocytes and their ligands is increased on endothelial cells of small postcapillary veins during neuroinflammatory processes. Infiltration of blood-borne leukocytes and immune cells at the site of brain injury has been found in experimental and clinical ICH (31–33). Currently, the management of acute ICH typically involves a comprehensive treatment approach that includes conservative therapy (strict blood press control, hemostatic drug administration, and prevention of complications) and surgical interventions (for cases where the hematoma’s mass effect leads to malignant intracranial pressure elevation, and even brain herniation) (34, 35). If patients are fortunate enough to survive the acute phase, chronic phase treatment typically involves the management of cognitive impairments (if present), active rehabilitation exercises (for functions such as language and motor skills), and long-term effective home care measures (36).

SAH is another subtype of hemorrhagic stroke. While it accounts for only 3–5% of stroke incidence, it carries a high mortality rate, poor prognosis and imposes a significant burden on individuals, families, and society (37, 38). SAH occurs when blood flows into the subarachnoid space following the rupture of intracranial blood vessels, with the rupture of intracranial aneurysms being the most common cause (39). The brain damage caused by SAH can also be divided into two stages: early brain injury (EBI) and delayed cerebral injury (DCI). EBI occurs within the first 3 days after the onset of SAH and is believed to be caused by transient global cerebral ischemia, the toxic effects of subarachnoid blood, and direct damage to brain tissue due to bleeding (40). The mechanisms of EBI are still not fully understood, but it is generally believed to be associated with oxidative stress, endoplasmic reticulum stress, BBB disruption, excessive inflammatory responses, and cell apoptosis (38, 40). An increasing body of evidence suggests that EBI is the primary cause of poor prognosis in SAH patients (41, 42). DCI is one of the hallmarks of SAH and is believed to be caused by the breakdown products of blood and inflammatory responses. It commonly occurs within 3–14 days after SAH and can lead to delayed neurological deterioration. The pathological basis of DCI may involve microcirculatory disturbances due to various factors, including large artery/microvascular spasms, microthrombus formation, and impaired venous outflow (43).

In summary, current treatment approaches have not yet significantly improved the prognosis of patients with hemorrhagic stroke. The limited understanding of the pathological mechanisms of hemorrhagic stroke constrains the exploration of more targeted therapeutic interventions. There is an urgent need for in-depth research into the pathogenesis of such diseases to guide the development of effective treatment strategies.

## 3 Overview of the TREM family

The TREM family is a recently discovered class of pattern recognition receptors predominantly expressed on the surface of myeloid cells. They can recognize one or multiple pathogen-associated molecular patterns (4). TREM genes in both humans and mice are situated within gene clusters on human chromosome 6p21.1 and mouse chromosome 17C (44, 45). With an immunoglobulin-like folded extracellular domain, TREM is a member of the evolutionarily conserved immunoglobulin superfamily (46). The human cluster includes NCR2 (encoding NKp44), TREM1, TREML4 (encoding TREM-like 4), TREML2, TREM2 and TREML1. The mouse cluster consists of Trem5, Trem4, Trem1, Trem3, Treml4, Treml2, Treml6, Trem2, and Treml1 (47). The TREM family has been found to be expressed on the surface of various cell types, including granulocytes, monocytes, tissue macrophages, macrophages, and dendritic cells (DCs). In general, the primary function of TREMs is to modulate the threshold and duration of myeloid cell responses. Research has revealed that TREMs have both positive and negative roles in regulating the activation and differentiation of myeloid cells and are associated with various pathological and physiological conditions, including inflammation, neurodegenerative diseases, bone remodeling, metabolic syndrome, atherosclerosis, and cancer (4).

### 3.1 Structure and function of TREM1

The earliest discovered TREM1 has been characterized as an amplifier of immune responses, capable of enhancing monocyte/granulocyte responses to microbial products (48). There are two isoforms of TREM-1: membrane-bound (mTREM-1) and soluble (sTREM-1). mTREM-1 is a type I transmembrane glycoprotein receptor composed of an extracellular immunoglobulin domain, a transmembrane region, and a cytoplasmic domain (49). The extracellular domain is primarily responsible for ligand binding, while the transmembrane region associates with the DNAX-activating protein 12 (DAP12) adapter protein through non-covalent bonds, transmitting signals within the cell (50). sTREM-1 is a glycosylated peptide with only one immunoglobulin-like domain. The ligands of mTREM-1 initiate the activation of the TREM-1 signaling pathway. Since this receptor lacks an immunoreceptor tyrosine-based activation motif (ITAM), it requires association with the ITAM-containing adapter protein DAP12 to transmit signals. The transmembrane structural domain of TREM-1 features positively charged lysine residues that form noncovalent bonds with the negatively charged aspartate residues found in the transmembrane region of the DAP12 junction protein. When DAP12 binds, tyrosine phosphorylation in the immunoreceptor tyrosine-based activation motif (ITAM) is facilitated by Src-family kinases. This, in turn, recruits the zeta-associated protein of 70 kDa (ZAP70) and spleen tyrosine kinase (SYK) (51), initiating a cascade of downstream signaling reactions. These reactions encompass the activation of pathways including phosphoinositide phospholipase C-gamma (PLCγ), phosphoinositide 3-kinase (PI3K), Janus kinase (JAK), and mitogen-activated protein kinase (MAPK). These pathways collectively induce processes such as Ca^2+^ mobilization, actin cytoskeleton rearrangement, and the activation of transcription factors. These transcription factors (including the nuclear factor of activated T cells (NFAT), signal transducer and activator of transcription 3/5 (STAT3/5)) are responsible for encoding the expression of cell-surface molecules, proinflammatory cytokines, and chemokines (52, 53). Little is known about the ligands that activate TREM1. Limited evidence suggested that high mobility group box 1 (HMGB1), PGN recognition protein 1 (PGLYRP1), heat shock protein 70 (HSP70), CD177, actin, and extracellular cold-inducible RNA-binding protein (eCIRP) may be ligands for TREM-1 (50, 54, 55).

### 3.2 Structure and function of TREM2

TREM2 is a transmembrane receptor belonging to the immunoglobulin superfamily of lectin-like proteins. Its ligands encompass various anionic molecules, freely present or bound to the cell membrane, including bacterial products, DNA, lipoproteins, and phospholipids (56). TREM2 is a crucial innate immune receptor predominantly expressed in myeloid cells, such as immature dendritic cells, osteoclasts, tissue macrophages, and microglial cells, among other cell types, both on the cell surface and within intracellular pools (4, 56, 57). TREM2 is an activating receptor associated with DAP12 and DAP10. It consists of a single V-type immunoglobulin domain, a short extracellular domain, a transmembrane helix, and a short cytoplasmic tail lacking any signaling or transport motifs (58). Research on mouse macrophages has shown that TREM2 binds to the adapter proteins DAP12 and DAP10 through reverse-charged residues in its transmembrane domain (58). Following the interaction of TREM2 with its ligands, these co-receptors become phosphorylated, initiating intracellular signaling mechanisms. DAP12, also known as Tyrosine Kinase Binding Protein (TYROBP), mediates the activation of SYK, whereas DAP10 promotes signal propagation by recruiting PI3K (59).

## 4 The role of TREM1 in hemorrhagic stroke

**Figure 1:**
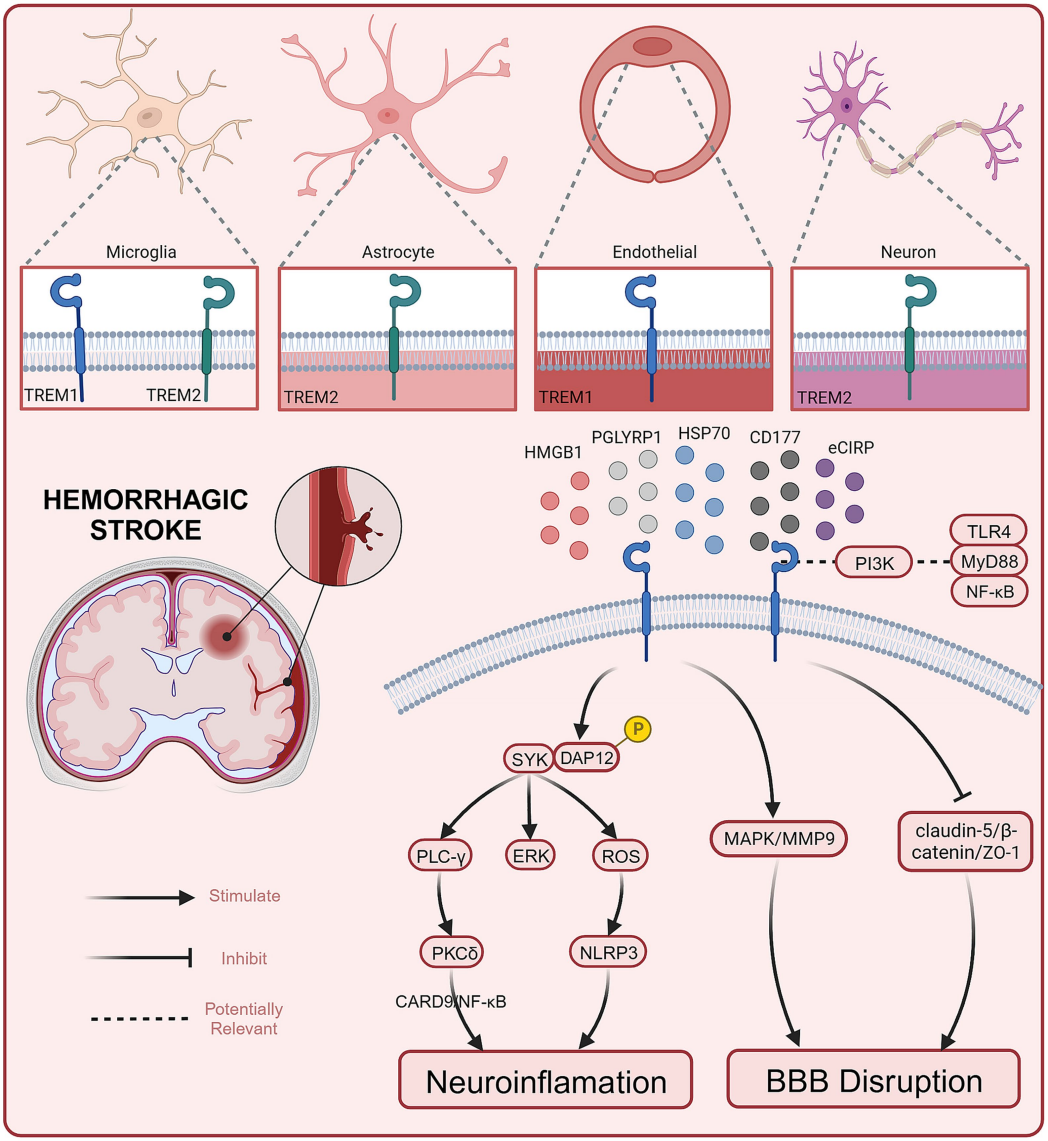
Role and potential mechanisms of TREM1 in hemorrhagic stroke.

To date, several preclinical studies have reported the role and potential mechanisms of TREM1 in hemorrhagic stroke (see Figure 1). Current evidence suggests that TREM1 mediates EBI after hemorrhagic stroke by engaging neuroinflammation and BBB destruction (summarized in Table 1).

**Table 1:** Established reports of TREM1/2 in hemorrhagic stroke.

Author	Time	Animal/cell	Model	Main results
Lu et al. (60)	2021	Mouse	ICH model via autologous blood injection	TREM1 enhanced neuroinflammation by modulating microglia polarization after ICH, and this regulation was partly mediated via PKC δ/CARD9 signaling pathway and increased HMGB1 activation of TREM1
Xie et al. (61)	2023	Mouse	ICH model via collagenase injection	TREM1 deteriorated BBB permeability via modulating the expression of inter-endothelial junction molecules after ICH, and this regulation is partly mediated by the SYK/β-catenin signaling pathway
Gu et al. (62)	2021	Human	ICH patients	Serum sTREM1 may represent a promising inflammatory biomarker for assessment of severity and prediction of END and poor outcome after ICH.
Xu et al. (63)	2021	Mouse; BV-2 microglia cell line	*In vivo* SAH model via endovascular perforation; *in vitro* SAH model were treated with 25 μM OxyHb for 24 h	TREM1 exacerbated neuroinflammation via NLRP3 inflammasome mediated pyroptosis after SAH. Blockade of TREM-1 can inhibit inflammatory responses, rescuing SAH outcomes.
Sun et al. (64)	2019	Rat	SAH model via endovascular perforation	TREM1 may participate in the pathogenesis of SAH-induced EBI via promoting p38MAPK/MMP-9 activation and ZO-1 degradation, while TREM1 inhibition attenuated the EBI severity obviously, providing a novel approach for the treatment of EBI
Chen et al. (65)	2020	Mouse	ICH model via intrastriatal injection of bacterial collagenase	TREM2 activation improved neurological functions and attenuated neuroinflammation and neuronal apoptosis after ICH, which was partially mediated by activation of the PI3K/Akt signaling pathway
Liu et al. (66)	2022	Mouse; BV2 microglia cell line	*In vivo* ICH induced by administration of collagenase-IV; *in vitro* ICH model performed with oxygen hemoglobin	TREM2 overexpression ameliorated ICH-induced neurological dysfunction, inhibited neuroinflammation, and attenuated apoptosis and brain edema
Zhou et al. (67)	2023	Mouse	ICH by stereotaxic injection of recombinant sTrem2 protein or by adeno-associated virus-mediated expression.	STREM2 acted as a negative factor against microglia/macrophage-mediated hematoma and related neuronal damage clearance
Sun et al. (68)	2017	Human	SAH patients	Elevated sTREM1 levels in CSF of Patients with Early SAH correlate with the extent and clinical severity of inflammation post-SAH
Wu et al. (69)	2021	Human patients; mouse	SAH model via endovascular perforation	Blockade of TREM1 suppresses inflammatory responses by attenuating proinflammatory subtype transition of microglia and decreasing the formation of neutrophil extracellular traps through interacting with SYK after SAH
Cao et al. (70)	2022	Rat	SAH model via endovascular perforation	TREM2 played a neuroprotective role and improved the short-and long-term neurological deficits by modulating neuroinflammation after SAH. Regulation of neuroinflammation by TREM2 after SAH is associated with elevated IRAK3 protein levels
Hu et al. (71)	2021	Human patients; Mouse	SAH model via endovascular perforation	TREM2 played a pivotal role in mediating microglial polarization after SAH, and the hyperactive TLR4 might potentially suppress the neuroprotective effect of TREM2 in the early phase of SAH

ICH, intracerebral hemorrhage; PKC, protein kinase C; SAH, subarachnoid hemorrhage; CARD9, Caspase Recruitment Domain Family Member 9; TREM, triggering receptor expressed on myeloid cells; HMGB1, high-mobility group box 1; BBB, brain–blood barrier; SYK, spleen tyrosine kinase; END, early neurologic deterioration; OxyHb, oxyhemoglobin; EBI, early brain injury; p38MAPK, p38 mitogen-activated protein kinase; MMP-9, matrix metalloproteinase-9; ZO-1, zonula occludens-1; PI3K, phosphatidylinositol 3-kinase; sTrem1/2, soluble TREM2; CSF, cerebrospinal fluid.

### 4.1 The role of TREM1 in ICH

TREM1 is an inflammation amplifier typically expressed on myeloid cells (72). Both in ICH and SAH, the secondary neuroinflammation is closely associated with the progression and prognosis of the diseases (20). Activated TREM-1 provides a docking site for SYK through the phosphorylation of DAP12, and SYK can activate the PLC-γ and extracellular signal-regulated kinase (ERK) pathways (73). It has been reported that PLC-γ can activate Protein Kinase C(PKC)-δ, and this activation has been demonstrated to stimulate microglia through the caspase recruitment domain family member 9 (CARD9)/NF-κB signaling pathway (74). In 2021, a study conducted by a Chinese team showed that serum sTREM1 levels were significantly higher in patients with ICH compared to healthy controls from admission to 24 h after admission. Furthermore, Gu et.al found that serum sTREM-1 concentrations were closely associated with National Institutes of Health Stroke Scale (NIHSS) scores, hematoma volume, blood leukocyte count, and serum C-reactive protein levels. By measuring serum sTREM-1 levels and three-month follow-up scores at prognosis (NIHSS score and modified Rankin scale (mRS) score), they found a significant increase in serum sTREM-1 concentration after ICH that may reflect the inflammatory response, hemorrhage severity and long-term functional prognosis. Therefore, sTREM-1 can be used as an inflammatory biomarker to assess the severity of ICH and predict early neurological function deterioration (62). TREM1 is primarily expressed in microglia/macrophages within the central nervous system (50). TREM-1 and HMGB1 expression increased after ICH, and the two proteins interacted to promote an inflammatory response. HMGB1 inhibitors alleviated neuroinflammation after ICH (60). In the EBI stage, microglia/macrophages polarize to the M1 phenotype and secrete pro-inflammatory factors involved in the process of EBI. Within the first 7 days after ICH, microglia/macrophages polarize to the M2 phenotype, aiding in hematoma clearance and brain injury repair (75). TREM1 can enhance neuroinflammatory responses by regulating the polarization of microglia/macrophages following ICH, causing them to shift toward the M1 phenotype. Selective knockout or pharmacological inhibition of TREM1 (LP17) reduces the secretion of pro-inflammatory cytokines after ICH, promoting the polarization of M2 phenotype. This, in turn, decreases brain edema and inflammatory responses after ICH and improves neurological symptoms in mice (60). In addition to microglia/macrophages, TREM1 has also been reported to be expressed in brain vascular endothelial cells (61). Another study found that early and delayed administration of LP17 significantly reduces brain edema and improves neurological behavioral performance 24 h after ICH. Specifically, TREM1 inhibition decreased BBB permeability by modulating the SYK/β-catenin signaling pathway and increasing the expression of β-catenin, claudin-5, and ZO-1 (61). In summary, TREM1 is expressed in different cell types in the CNS and plays a dual “harmful” role in promoting inflammation and exacerbating BBB disruption following ICH. Targeted interventions to modulate TREM1 may have a positive impact on improving the prognosis of ICH. Elevated serum levels of TREM1 in patients with ICH, although awaiting further confirmation in a multicenter study, suggests a basis for future clinical studies to be conducted.

### 4.2 The role of TREM1 in SAH

Extensive research suggests that EBI is a significant contributor to poor outcomes in SAH, and EBI is closely associated with neuroinflammation (41, 42). Sun et al. found a substantial increase in sTREM-1 levels in the cerebrospinal fluid (CSF) of SAH patients. The extent of this increase was closely related to the intensity of inflammatory responses (TNF-α, IL-6, white blood cell count, and CRP levels) and the severity of the disease (Glasgow coma scale (GCS), World Federation of Neurosurgical Societies (WFNS) scale, Hunt and Hess scale). This suggests that TREM-1 may be involved in initiating and amplifying the inflammatory cascade processes in EBI following SAH (68, 76). Sun et al. reported that TREM1 is primarily expressed in microglia/macrophages and brain vascular endothelial cells, consistent with previous reports. Additionally, they demonstrated that the expression of TREM-1, TLR4, MyD88, and NF-κB p65 increases following SAH. Inhibiting TREM-1 can alleviate EBI associated with TLR4/MyD88/NF-κB inhibition. Inhibiting TLR4 can prevent TREM-1 induction and improve EBI. Furthermore, the levels of sTREM-1 in SAH patients are positively correlated with Hunt-Hess score (76). All of this evidence suggests a potential role for TREM1 in mediating early brain injury after SAH. Previous research has also found the involvement of PI3K in both the TREM-1/DAP12 pathway and the TLR4/MyD88 pathway. PI3K seems to play a crucial role in both TREM-1 and TLR4 signal transduction, leading to the synergistic release of ROS (77). This suggests that there may be a connection between the TREM-1 pathway and the TLR4/MyD88/NF-κB signaling pathway (78). Wu et al. further explored the role of TREM1 in neuroinflammation following SAH and its potential mechanisms (69). Mechanistic studies have shown that TREM1 activates a downstream proinflammatory pathway through interaction with splenic tyrosine kinase (SYK). Pharmacological inhibition of TREM1 attenuates the proinflammatory subtype switching of microglia/macrophages and the formation of extracellular traps in neutrophils, which reduces neuroinflammation after SAH, decreases brain water content, reduces neuronal damage, and ultimately attenuates neurological deficits.

In addition, the role of TREM-1 in BBB disruption following SAH has also been explored. The BBB comprises a structural barrier formed by tight junction-associated proteins, including ZO-1. The degradation of ZO-1 disrupts tight junctions and leads to BBB breakdown (79). Previous studies have suggested that TREM-1 is involved in the pathogenesis of BBB disruption by activating MAPK and degrading ZO-1. Meanwhile, TLR4, through the MyD88 pathway, leads to NF-κB induction and MAPK activation. Subsequently, MAPKs participate in the degradation of ZO-1 and the breakdown of the BBB following SAH (64, 80). Therefore, TREM-1 and TLR4 may form a positive feedback loop by activating MAPKs, exacerbating EBI. In another study, TREM-1, the NACHT, LRR, and PYD domains-containing protein 3 (NLRP3) inflammasome, cleaved caspase-1, mature IL-1β, and mature IL-18 increased expression following SAH. The NLRP3 inflammasome can be activated through active oxygen production dependent on SYK, and the TREM-1/DAP12 signaling cascade can recruit SYK. This suggests that TREM-1 may activate NLRP3, triggering an inflammatory response and leading to the death of microglia/macrophages through SYK (63, 81). Combining *in vivo* and *in vitro* research, Xu et al. found that activated TREM-1 could trigger pyroptosis in microglia and exacerbated neuroinflammation after SAH through activation of NLRP3 inflammasome (63). All evidence suggests that elevated TREM1 after SAH is indeed involved in the progression of early brain injury, at least in part by exacerbating neuroinflammation and BBB destruction. Targeting TREM1 is expected to exert a neuroprotective effect as one of the potential therapeutic options for patients with SAH. However, before that can happen, more precise mechanistic pathways and extensive and effective clinical studies must first be addressed.

## 5 The role of TREM2 in hemorrhagic stroke

In recent years, several teams have investigated the role of TREM2 in hemorrhagic stroke. Although there are few reports, limited evidence suggests that TREM2 exerts some neuroprotective effects in hemorrhagic stroke (Table 1).

### 5.1 The role of TREM2 in ICH

In animal models of neurodegenerative diseases such as Alzheimer’s disease, Parkinson’s disease, multiple sclerosis, and ischemic stroke, overexpression of TREM2 has been reported to suppress neuroinflammation and exhibit significant neuroprotective effects (9, 82–84). In recent years, several studies have explored the role of TREM2 in ICH. Chen et al.’s research suggests that TREM2 expression is significantly upregulated in microglia/macrophages, astrocytes, and neurons following ICH. Intranasal administration of the TREM2 ligand COG1410 can upregulate TREM2, PI3K, phosphorylated Akt, reduce brain edema, suppress microglia/macrophage activation and neutrophil infiltration, and inhibit neuronal apoptosis in the perihematomal region after ICH. Ultimately, this leads to improved neurological function. These conclusions are further validated by the fact that knockdown of TREM2 resulted in worsening neurologic dysfunction (65). Another study reported similar neuroprotective effects of TREM2 in ICH (66). An *in vivo*/*ex vivo* study by Liu et al. demonstrated that TREM2 attenuates neuroinflammation and neuronal apoptosis during the acute phase of ICH by negatively regulating the TLR4 signaling pathway and ultimately ameliorates ICH mice’s neurological dysfunction. The latest study delves deeper into the potential role of TREM2 in hematoma dissipation (67). Their findings suggest that soluble TREM2 (sTREM2) hinders hematoma resolution and affects the recovery of motor and sensory functions. Specifically, sTREM2 is a negative regulator inhibiting microglial/macrophage-mediated clearance of hematoma and associated neuronal damage. This may partially explain the impaired erythrophagocytosis following ICH. The evidence above suggests that TREM2 is involved in regulating neuroinflammation and hematoma clearance following ICH by modulating the activation and infiltration of immune cells, particularly microglial cells and macrophages. Further studies could address the above different roles of TREM2 in detail, for example, whether there is crosstalk between neuroinflammation and hematoma clearance mechanisms. It has been shown that TREM2 is expressed in the astrocytes, but whether it is involved in BBB destruction has not been further reported. In addition, TREM2 is also expressed in neurons, but its specific function is unknown. Extensive and in-depth studies based on *in vivo* and *in vitro* experiments with the help of multi-omics analysis, including single-cell sequencing, are needed to answer these questions.

### 5.2 The role of TREM2 in SAH

There is limited evidence regarding the role of TREM2 in SAH, but Hu et al. conducted a study on this (71). The results indicated that TREM2 is expressed in microglia/macrophages and increases delayed after SAH. TREM2 knockout led to activation of microglia/macrophages, increased pro-inflammatory factors and deterioration of neurological function. As the evidence suggested, TLR4 knockout increased TREM2 expression while improving neuroinflammation and neurological function. The authors speculate that the neuroprotective effect of TREM2 may be suppressed by the exaggerated TLR4 expression in the early stages of SAH, which could explain the delayed increase in expression. One year later, Cao et al. found that elevated TREM2 could regulate neuroinflammation by increasing IRAK3 expression and exert neuroprotective effects after experimental SAH in rats (70). This complements the findings of Hu et al. by clarifying the upstream and downstream regulators of TREM2 after SAH, respectively. Further in-depth studies are expected to clarify the mechanism of TREM2 pathway in regulating neuroinflammation after SAH. Limited evidence suggests that, unlike the “deleterious” role of TREM1 in SAH, TREM2 appears to be neuroprotective. It is intriguing why structurally similar TREM receptors exhibit almost opposite functions, and further in-depth studies will help us better understand the underlying mechanisms.

## 6 Summary and prospects

In summary, current evidence suggests that the TREM1 signaling pathway is involved in the inflammatory response and BBB disruption in hemorrhagic stroke and ultimately exacerbates secondary brain damage. Targeting the inhibition of the TREM1 signaling pathway, such as using the TREM1 inhibitor LP17, can mitigate the inflammatory response and blood–brain barrier disruption, improving neurological function in animal models of ICH and SAH. Conversely, preclinical studies suggest a neuroprotective role for TREM2. As the evidence indicates, TREM2 can inhibit microglial/macrophage activation and neutrophil infiltration, reduce brain edema, and inhibit neuronal apoptosis in the perihematomal region. TREM1 and TREM2 have similar structures but different functions. This difference is well summarized by Zhang et al. (50). The potential roles of TREM1 and TREM2 in neuroinflammation and BBB disruption provide new insights into targeted therapies for ICH and SAH. Moreover, serum sTREM1 was elevated within 24 h after ICH (62) and positively correlated with Hunt-Hess scale in patients with SAH (76). Further studies need to explore its potential as a biomarker for hemorrhagic stroke.

It is well known that during acute stress, microglia/macrophages release a range of inflammatory mediators, including cytokines and chemokines. These acute-phase responses are often thought to favor neuronal survival, attenuate secondary damage and ultimately help restore cellular homeostasis (85, 86). During chronic inflammation, the continued release of inflammatory mediators by microglia/macrophages causes sustained oxidative stress and is therefore considered to be detrimental (87, 88). Studies have focused on TREM1/2 in the acute phase of ischemic stroke; what role TREM1/2 fills in the chronic phase is largely unknown, which is a direction for future research. Notably, most published ICH studies have been unable to distinguish between macrophages and microglia (which we refer to here as microglia/macrophages) because the two cell types are difficult to distinguish *in vivo* (89, 90). With the help of more specific cellular markers (e.g., Tmem119 for microglia) and multichannel flow cytometry, these two cell types are expected to be rigorously distinguished in the next explorations (91, 92). On this basis, it will be fully explored whether microglia and macrophages play different roles (if any) in ICH.

In addition, the above studies inevitably have other limitations. For instance, the exact roles of TREM1/2 in other cell types in the central nervous system (e.g., TREM2 in astrocytes and neurons) have not been fully elucidated. Additionally, the current research is primarily preclinical and mainly involves rodent animals. The roles of TREM1/2 in hemorrhagic stroke need further validation in higher primates and clinical studies. Finally, elucidating the mechanisms of action and signal regulation between TREM1/2 and their respective ligands will help us understand their biological properties and provide a solid theoretical basis for developing potential future treatment options.

## Author contributions

YK: Conceptualization, Funding acquisition, Methodology, Resources, Writing – original draft, Writing – review & editing. DW: Investigation, Visualization, Writing – original draft. XJ: Formal analysis, Writing – review & editing. YL: Supervision, Writing – review & editing. HX: Data curation, Supervision, Writing – review & editing.
